# Challenges in Palliative Care in Latin America: A Narrative Review

**DOI:** 10.7759/cureus.60698

**Published:** 2024-05-20

**Authors:** Joseph Pergolizzi, Jo Ann K LeQuang, Morgan Wagner, Giustino Varrassi

**Affiliations:** 1 Anesthesiology - Pain Medicine and Critical Care Medicine, NEMA Research, Inc., Naples, USA; 2 Scientific Communications, NEMA Research, Inc., Naples, USA; 3 Entrepreneur Program, NEMA Research, Inc., Naples, USA; 4 Pain Medicine, Paolo Procacci Foundation, Rome, ITA

**Keywords:** cancer in latin america, latin america, pain management at the end of life, end of life and hospice care, supportive and palliative care, hospice and palliative care, palliation

## Abstract

In “graying” populations with extended lifespans and survivable forms of cancer, palliative services become increasingly important but may be difficult to introduce into public discourse, public policy, and healthcare systems. Latin America (LATAM) faces many challenges as it introduces and, in some cases, develops its palliative care programs; though the challenges faced here are in many ways universal ones, LATAM approaches may be unique and based on the region’s specific culture, politics, and economics. This narrative review based on a literature search identified 10 main themes that can be interpreted as challenges and opportunities for palliative care in LATAM. These challenges are integrating palliation into healthcare systems; public policy and funding; therapeutic obstinacy; changing demographics; access to services; analgesia; the role of religion, spirituality, and folk medicine; social determinants of palliative care; low health literacy; and limited clinician training. Some of the LATAM nations have palliative programs and palliative care training in place while others are developing these systems. Integrating this care into existing healthcare and reimbursement systems has been a challenge. A notable challenge in LATAM is also access to care since palliative programs tend to cluster in metropolitan areas and create hardships for rural citizens to access them. The better-defined role of familial caregivers and telehealth may be important factors in the expansion of palliative care in LATAM and beyond.

## Introduction and background

Canadian academician and physician, Balfour Mount, MD, coined the term “palliative care” in 1974 to describe work that commenced in 1959 when a British nurse named Cicely Saunders wrote her first article on managing terminal patients [1]. Palliative care is a multidisciplinary approach to the physical, medical, social, psychological, and spiritual needs of patients faced with life-limiting illnesses, including those at the end of life [1]. At first, such work focused primarily on cancer patients but the emphasis in the middle of the twentieth century was on curing cancer, not managing it or helping patients at the end of life. Indeed, many terminal cancer patients were discharged home with dire words from the clinical team, “There is nothing more we can do” [1]. In Europe, this led to the hospice movement, but it has since expanded to care for life-altering illnesses, and by the 1970s, palliative programs were introduced around the world [1].

A key focus of palliation was pain control, which was first addressed as a global public health topic by the World Health Organization (WHO) with its iconic pain ladder in the 1980s for the treatment of pain in adult cancer patients [2]. Cancer has since emerged as a healthcare threat in low- and middle-income nations and has particularly challenged the many nations of Latin America (LATAM) [3]. Today palliation has become an important topic in global healthcare, with variable progress from nations and regions around the world, particularly in the field of clinical training, clinical readiness, and pain management. Challenges face each region and nation, sometimes universal but sometimes specific to the local culture, religion, history, and healthcare resources. In LATAM, the availability of opioid analgesics for cancer and other forms of severe pain has been limited, often for political rather than medical reasons [4, 5]. Nevertheless, the LATAM nations have made remarkable strides in palliative programs since 2000 but local, regional, and global challenges remain.

The diverse nations of Latin America (LATAM) include Mexico, Central America, South America, and the Caribbean countries. While this is a vast, populous, and culturally heterogeneous region, there are important sociocultural, political, and medical similarities and distinctions in terms of how palliative care is viewed, implemented, and perceived. Like all regions, LATAM faces considerable challenges in forging palliative policy and delivering palliative care, and many of these specific issues have broad implications for palliative care as a whole. Guidelines for palliative care and national quality indicators for palliation are lacking in LATAM and may be impeding the development of palliative programs. While palliative care continues to expand in LATAM, it remains under-developed in contrast to Europe and North America [6]. However, LATAM faces unique challenges and has unique opportunities.

Palliation may sometimes be seen as reversing the traditional treatment paradigm of saving and extending life at all costs. Comfort is prioritized over treatment. For some clinicians, palliation seems like “giving up” and conflicts with their deeply held ethical and professional convictions. Despite advanced levels of care and breakthroughs in medical treatments, death remains difficult to predict and even difficult to discuss. At the end of life, many significant influences converge: science, religion, medicine, spirituality, family, and law [7]. Palliation demands that the patient and family travel a difficult path, guided by clinicians who often have little training, guidance, or resources to help guide them. While palliative care is seen as a healthcare service, palliation requires a multidisciplinary approach to care that often exceeds the skills and abilities of clinicians.

In reviewing palliative care in the large and heterogeneous region of LATAM, certain key issues emerged as fundamental obstacles to better and more comprehensive palliation programs. While the research was limited to LATAM nations, it is hoped that a discussion of these barriers to better palliative care offer some universal insights. Though there remain cultural, political, religious, and economic distinctions among regions of the world and among nations within those regions, the core issues raise the fundamental points to consider.

Methods

In October 2023, the authors searched PubMed for “Latin America palliative care” and retrieved 133 results. A search for “Central America palliative care” resulted in 76 articles. “Caribbean palliative care” yielded 129 results. “Palliative care Mexico” produced 485 results. There were considerable duplications in these searches. The inclusion criteria for articles were reviews, clinical studies, and randomized clinical studies written in English and published in or after the year 2000 and addressing specific issues in palliative care in LATAM. The exclusion criteria were articles published before 2000, articles not in English, articles about pediatric palliation, case studies and correspondence, and articles that did not address palliative care in LATAM. This resulted in 75 articles. The content of the articles was categorized into 10 main themes which appear as subheadings in the latter portion of this paper. These themes were determined by the authors as an attempt to better distill the large amount of content into meaningful discussions for the broader issues of palliative care.

Note that LATAM is defined here as the geographical region that encompasses the Caribbean nations and all nations of the Western Hemisphere south of the United States, although other definitions describe the region linguistically as those parts of the Western Hemisphere where Spanish, Portuguese, or French are spoken. Haiti and Cuba were excluded from our study as being anomalies in LATAM. Haiti is a French-speaking nation with a distinct different culture to most LATAM countries and Cuba has a markedly different political system that affects how it manages national healthcare. There is limited data from these two nations about palliation in those countries.

## Review

The Latin American Association for Palliative Care (ALCP) oversees palliative services and has identified over 900 palliative care services in LATAM [8]. Palliative services are expanding in LATAM [9], but only about 7% of LATAM citizens who need palliative care have access to these services, mainly available to cancer patients in large cities [10]. From the literature on palliation in LATAM, 10 themes emerged that have universal or near-universal application; namely, integrating palliation into healthcare systems; public policy and funding; therapeutic obstinacy; changing demographics; access to services; analgesia; the role of religion, spirituality, and folk medicine; social determinants of palliative care; low health literacy; and limited clinician training.

The integration of palliative care into healthcare systems

A healthcare system is based on its nation’s underlying political and economic structure as well as national political goals and the nation’s political resolve [11]. Since LATAM is not a homogenous region with similar political structures, economies, and national objectives, it is clear that its healthcare systems will diverge as well, affecting how nations define palliative care. Despite this lack of clear definition, palliative care has been advanced as a human right [12]. The urgency to achieve better palliative care is recognized, but guidance is scarce. In 2020, the World Health Organization (WHO) issued a fact sheet on palliative care and expanded its definition beyond care for people with cancer or AIDS to those with life-threatening diseases or conditions of all types [13].

Many LATAM healthcare systems are in transition or undergoing reform. LATAM nations often operate parallel public and private healthcare systems [14] and sometimes palliative care is offered to those with private, but not public insurance. Thus, LATAM nations will approach healthcare, including palliation, with their own national perspectives, resolves, and goals; palliative care may be prioritized differently in different nations. This underscores that all nations pursuing palliative care must find ways to build high-quality care into an existing healthcare system, ideally creating a robust system providing services in an equitable and accessible fashion.

Public policy and funding

Developing a national plan and public policy for palliation is a crucial initial step. From 2012 to 2020, the number of LATAM nations with a national plan for palliative care doubled from five to 10 [15]. Public policy must integrate palliation into the national healthcare and/or reimbursement systems, rather than relegating it to an add-on service or some sort of luxury care. When palliative services are not directly incorporated into public policy, it can create difficult or even insurmountable barriers for palliative care to overcome [16]. WHO and the World Palliative Care Alliance have long advocated for incorporating palliative programs into existing healthcare systems and policies [17]. In LATAM, four nations have adopted legislation related to palliation: Colombia, Mexico, Panama, and Uruguay [18]. Seven LATAM nations have national palliative care plans and 13 have a cancer palliation plan [18].

Low-income nations may have such limited resources that a full range of palliative services is not possible. The fundamentals of palliative care as recommended by the WHO are pain relief, symptom control, and spiritual or psychological support [19]. The strong family traditions prevalent in LATAM make home care at the end of life feasible, which can cover local service gaps [19]. Home care by relatives may be a necessity in some parts of LATAM where there are few beds dedicated to palliative care and the public health system offers no home-health options for palliation [20]. Many nations depend on unpaid family members for palliation, and these uncompensated individuals are not often considered in calculating the costs of palliation. Policy remediation may offer these family caregivers meaningful benefits such as guaranteed time off from employers or tax benefits to help cover expenses related to buying medicines or equipment. Although seldom offered in LATAM, supplemental services, from housekeepers to temporary home healthcare workers, can help lighten the burden placed on unpaid family caregivers and make familial care feasible [17]. 

Opioid analgesic consumption is low to extremely low in LATAM overall, in part because of public policy, restrictive regulations, and government concerns about opioid abuse [8]. For instance, oral morphine is not expensive and is considered an essential medicine [21], but its use may be limited by legal and social concerns more than economics. Thus, palliation policies must also address the frequently contentious topics of opioid availability, opioid distribution, and prescribing regulations.

Palliative care is not universally recognized in LATAM as a medical specialty, so such services may not be fully reimbursed or reimbursed at all. In some LATAM nations, charitable institutions are expected to pick up the slack for palliative care, including recruiting volunteer caregivers. Wealthier families may be able to pay out of pocket for palliative services, but most families are not able or are unwilling to pay out of pocket for such care [20]. Many LATAM residents may not be aware that palliative services exist, much less how to access them.

Needs assessment protocols may be helpful in better identifying and defining palliation needs in LATAM [22]. An epidemiologic approach to needs assessment would evaluate the size of the need, the prevalence of the problem, and all local services available, including how effective and cost-effective such services are. These three data points (need, prevalence, local services) can easily expose gaps in palliative care or areas where service provisions do not match actual needs. A needs assessment for palliation exceeds the expertise of healthcare professionals, requiring the expertise of public health experts, sociologists, and epidemiologists [22]. Failure to meet the need for palliation can be expensive. A Brazilian study of 111 patients in their last 30 days of life with hematologic malignancies found high rates of healthcare utilization, including high rates of emergency department visits (67%), intensive care hospitalizations (56%), blood transfusions (45%), and medical imaging services (59%). Overall, these patients received suboptimal care that was overly expensive to the healthcare system [23]. A targeted and tailored palliative care program even on a small scale may have been able to meet patient needs without unnecessary emergency hospitalizations.

Fragmented healthcare services in LATAM sometimes operate as “islands,” detached from each other, which causes them to be overlooked in national health policies and government funding. For example, laws on specific topics that may be relevant to palliation, such as access to opioid analgesia or paid leave for family caregivers, may fail to consider the needs of palliative programs [19]. There are also historical reasons shaping LATAM palliative programs. In the United States, palliative care grew out of the hospice movement, but in LATAM, palliative care emerged from specialized hospital services, often related to oncology. As such, palliative care in LATAM is often viewed as a physician-led service limited to specific types of cancer care. Nurse practitioners, who play an important role in palliation in other countries, play only a limited role in LATAM palliation [24].

Therapeutic obstinancy and decision-making

Therapeutic obstinacy refers to the initiation or prolongation of medical treatments that cause suffering and distress to a patient with no reasonable expectation of recovery [6]. In a survey among LATAM physicians, 37.7% stated they believed that some physicians exhibit therapeutic obstinacy and continue to recommend cancer treatments for incurable diseases, even when they were probably going to be ineffective [25]. Aggressive interventions and treatments of last resort may be viewed by some in medicine as heroic and supporting the patient’s “fight” for survival, but Spanish-language descriptions of “suitable therapeutic efforts” (adecuación esfuerzo terapéutico) have been proposed as an alternative and may be a strong foundation for palliative care efforts. While difficult to translate exactly, the phrase “suitable therapeutic efforts” suggests that care at the end of life should be what is appropriate for the patient rather than what is appropriate for the disease [7]. It approaches the English-language concept of patient-centric holistic care for palliative patients.

In many parts of LATAM, the healthcare system is paternalistic rather than collaborative, refuting the United States and European models of “shared decision-making” between clinical team and patient. Nevertheless, a growing sense of patient autonomy is emerging in LATAM, and communication between patients and providers has become more open [26]. However, patients, their families, and healthcare authorities in LATAM still regard physicians as primary decision-makers who, in some cases, have been known to go to the extent of withholding diagnoses and prognoses from their patients. From a survey and semi-structured interviews at a single neurological center in Peru, physicians and nurses had divided opinions as to who should inform a patient about an adverse prognosis, if the patient was to be informed at all. There is still a prevailing notion that, in some cases, it may be kinder to keep bad news from the patient. This paternalistic approach may be accepted in palliative care when patients have cognitive deficits or neurologic disease processes that make their participation in their own care difficult [27]. Shielding the patient from a truthful prognosis is not always the objective of the physician; sometimes the family may wish to protect the patient from upsetting news and will make efforts to keep this information from the patient, even if the patient wishes to learn the truth [27].

Palliative radiotherapy is an example of a treatment that does not prolong life but may bring comfort. In an online survey of palliative physicians in Chile, it was found that almost all respondents considered palliative radiotherapy useful (98%) but only 43% self-reported that they had a good knowledge of it [28]. Some of the main barriers to its use were reticence by the patient and/or family to undergo radiation treatments only for the sake of comfort [28].

The legal rights of patients in palliation are not well defined in LATAM. An advanced directive (AD) is a legal document spelling out decisions about end-of-life care prepared by patients while they have the mental capacity to make such decisions. ADs originated in the United States but have been adopted by other nations, including a few countries in LATAM [27]. The strength of the family and religious convictions may cede decision-making to the family as the patient nears death, relegating the dying person to an honored but passive role [29]. Based on a survey of 387 caregivers for palliative patients (100 in Chile, 99 in Argentina, 97 in Guatemala, and 91 Hispanics living in the United States), most caregivers preferred a shared decision-making model with the patient while they were able to make decisions [29]. As might be expected, giving the patient a more active role in decision-making was more common among Hispanics in the United States than in LATAM nations [29]. A survey of 182 cancer patients in LATAM and among Hispanics in the United States found that 48% wanted shared decision-making with their caregivers, 31% preferred to make their own decisions, while 21% wanted their caregivers to make decisions. Most patients (92%) stated they desired truthful information about their diagnosis and 94% wanted to know their prognosis [30].

While palliative care is typically perceived as care at the end of life, the WHO and other groups suggest that, when available, palliation services be included upon diagnosis of a life-threatening illness or incurable condition. This aligns well with the Spanish term for this process, "acompañar", which means to accompany or travel along with the patient on the journey [25]. The early integration of palliation into care for serious illness helps balance the risks of treatments against the patient’s quality of life and may better meet the goals of the patient and family.

An important cultural consideration in LATAM is the relationship between physician and patient or, by extension, between the clinical team and the patient within the context of family. LATAM healthcare tends to emphasize a personal relationship between the clinical team and the family, allowing for warmth and support but often delaying decision-making as the wishes of all family members must be solicited, discussed, and taken into account. LATAM families tend to be organized along patriarchal lines with deep respect afforded to certain members, often based on their age and authority within the family. Palliative care decisions must often be made or at least endorsed by these respected individuals [18]. Such collaborations strengthen family bonds but may delay care, avoid important decisions, and may not always reflect the patient’s choices.

Since the traditional family represents the bedrock of LATAM culture, caregiving often falls to family members who view their role as both a crucial life-sustaining function as well as a noble service. These notions should not overlook the fact that caregiving can be draining work that may exceed the physical, emotional, or psychological capacity of one individual. Palliative services in all nations benefit from these unpaid caregivers, whose work should be recognized and burdens alleviated when possible, for example with simple interventions that allow caregivers rest and respite [18]. Such interventions may include visits from home healthcare workers or services such as meal deliveries or running errands. The cultural context of caregiving must not be overlooked: however overwhelmed and exhausted caregivers may feel, they will likely view caregiving as an important and nearly sacred duty. Thus, efforts to alleviate the workload must not diminish the responsibility of the caregivers or detract from their honored role.

Changing demographics

The population in LATAM is aging; over 100 million residents are currently over the age of 60 and it is anticipated that half of this number will live past 80 years [18]. The population growth rate in LATAM is about 1.4%, slightly above the global rate of 1.2%, but high migration rates are reducing the younger population at the same time that extended life expectancy is increasing the older population. Demographic transitions affecting LATAM include decreased fertility rates, higher rates of infant survival, and longer life expectancy, which rose from approximately 51.4 years for men in 1950-54 to 71.5 years in 2000-2004. It is expected that by 2050, 25% of LATAM will be over the age of 60, and 5% will be among the oldest in the senior population, defined as ≥ 75 years. More than half of LATAM is expected to be ≥ 40 years old by 2050. These demographic shifts, which also affect the United States, Western Europe, Japan, and China, may require structural changes to social assistance and healthcare programs [31]. The aging population will increase the demand for palliative care.

Further increasing the demand for palliation is the increasing number of cancer patients, as cancer rates remain a leading cause of death in most LATAM countries [16]. Approximately 40% of LATAM palliative care patients have cancer [18]. It is estimated that three-quarters of all cancer patients in the world are terminal at the time of first presentation [17]. In many developing nations, cancer patients often present with late-stage disease because of limited access to medical services, economic barriers, and/or low health literacy [17]. Treatment abandonment remains common in oncology, even for childhood cancers, and poses a major challenge to healthcare systems and palliation efforts. Even when there is a commitment to long-term treatment, the costs, distance to clinics, travel requirements, work pressures, and other conditions conspire to derail treatment adherence [32]. As a result, many cancer patients in LATAM never receive palliative services.

Access to services

In South America, only about 10% of those who need palliative services have access to them, and 97% of those services are available only in larger cities [20]. LATAM is home to the three largest cities in the Western hemisphere, São Paulo, Mexico City, and Lima, but it also has vast areas of sparsely populated rural regions, creating tension between metropolitan areas with state-of-the-art clinics and isolated regions with minimal healthcare services [9]. A case in point is Bolivia, where palliative care has lagged behind much of the rest of LATAM. Ongoing efforts are offering more and more multidisciplinary palliative services to patients, but these services are being developed in the cities while those outside the major population centers have fewer options [33].

Telehealth may be able to help bridge the gap. Brazil has developed a large telehealth-based home cancer care program which provides a variety of services, including palliative care [34]. This model depends on family caregivers, who may experience a learning curve in mastering the app-based system. Furthermore, the role of the clinical staff in telehealth home palliation sometimes becomes unclear [35]. Nevertheless, telehealth options may become important as palliative care options and awareness increase.

Analgesia

In 1986, WHO advocated opioid analgesia for cancer pain patients and its iconic “pain ladder” model for treating cancer pain is still in use today [19]. Pain control is one of the pillars of palliative care. More than just a feeling, pain is a biopsychosocial phenomenon contextualized within the patient’s larger framework of culture, family, faith, and society [36]. The Spanish word for pain, “dolor,” encompasses a sense of sorrow, grief, and loss that the English word “pain” lacks [37]. Family traditions, spiritual background, and social norms all color how pain may be viewed. Pain may be viewed as natural and expected; suffering may be elevated to a religious virtue; and stoic endurance may be considered a sign of strength. Thus, some patients may hesitate to ask for pain relief while others may want their pain controlled but refuse opioids for fear of addiction. Family caregivers are not generally knowledgeable about pain relief and may not be reliable observers for pain assessments; some patients may conceal their pain from their families [38].

The restrictions on opioids in many LATAM nations along with cultural attitudes about opioids pose a challenge for pain control in palliative care [39]. It is not surprising that opioid consumption in LATAM falls below the global average and far below consumption rates in the United States and Europe [39].

A survey about opioids was administered to 436 healthcare professionals in 17 LATAM nations from August 2019 to October 2020 [40]. Approximately half of the respondents worked in palliative care with a mean experience of 10 years (range 1 to 35 years). The primary barriers to prescribing opioids were culturally anchored beliefs about pain, patients’ fear of addiction, and financial constraints. Clinicians reported a lack of training in opioid prescribing, inadequate pain assessments, and fear of creating opioid dependence. In some cases, physicians offered to prescribe opioids, but patients refused them. In certain but not all parts of LATAM, obstacles to the use of opioids were limited dispensaries, periodic shortages, supply chain failures, and regulations that limited access to these drugs, even when lawfully prescribed. These barriers varied by country, with major barriers reported in Mexico and fewer obstacles in the far south of South America [40].

Opioid use is increasing in LATAM. From 2012 to 2020, the morphine milligram equivalence (MME) of dispensed opioids increased from 6.6 to 7.1 MME per capita [41]. LATAM is heterogeneous in terms of opioid consumption, with some nations consuming what might be termed “moderate” levels, such as Argentina, Brazil, Chile, Colombia, Mexico, Costa Rico, and most of the Caribbean nations, but countries such as Guatemala, Bolivia, and Honduras reporting “low” levels. Data on opioid consumption are not available for some nations and are presumed low [8]. Most countries set a 30-day maximum number of days per prescription, but a few nations had shorter times: Ecuador (three days) and Argentina, Costa Rica, Dominican Republic, Jamaica, and Peru (10-15 days). Bolivia’s prescribing restrictions were based on dose rather than days. In Bolivia, Paraguay, Uruguay, and St. Lucia, prescription duration was left to the discretion of the physician [8].

While educational interventions in the safe and effective prescribing of opioid analgesics to palliative patients are generally helpful in terms of improving clinician knowledge, they do little to alleviate ossified legal, regulatory, economic, and administrative barriers [42].

The role of religion, spirituality, and folk medicine

Spiritual practices, rituals, and prayers form an important coping mechanism for palliative patients and their families all over the world, and religion is an important aspect of modern life throughout LATAM. Patients and their families typically seek spiritual guidance when dealing with life-threatening illnesses, and clinical teams can benefit by forming alliances with local religious authorities and communities to help guide palliation services [18]. In a survey of physicians in LATAM, only 29.2% felt confident dealing with the spiritual needs of their patients, with those who had specific training in palliative care more likely to express confidence [25].

Religion and spirituality are sometimes used interchangeably, but there are important distinctions. Spirituality in the current definition may be defined as the way individuals seek and express meaning and purpose, how they experience their connections to other humans and to nature, and what they regard as sacred [43]. Spiritual people may or may not have an affiliation to a church or other religious body. Some spiritual people may even report themselves as being atheists but feel a sense of universal connection to nature and other people. Religion, on the other hand, refers to an organized system of beliefs and often involves membership in an organization.

In the United States and Western Europe, chaplains act as generalists in spiritual care, but the Spanish word for a chaplain, "capellan", strongly suggests a religious affiliation. Therefore, the term spiritual care "specialist" is preferred by some in LATAM since it includes those who those who operate both within or outside of a religious system.

Although spirituality has been shown to help dying patients cope more positively with their condition, spiritual pain has been identified as a source of psychological distress in the final days of life. Spiritual pain differs from physical pain in that it lacks a physical focus. Spiritual pain cannot be easily quantified on a pain scale or even described well by the patient. In palliative patients, spiritual pain often revolves around extracting meaning from the patient’s approaching death. Recent research on this topic has proposed a definition that spiritual pain is discomfort triggered by the person’s relationship to God or a higher power [44].

In a survey of 315 palliative patients in Chile, Guatemala, and among Hispanics in the United States, most considered themselves as being religious or spiritual (89% and 97%, respectively) and 98% said that their beliefs helped them cope with their medical condition, but 60% did not feel their clinical team supported their spiritual/religious needs [37]. Palliative patients may have unmet spiritual/religious needs, to which the clinical team may be oblivious. In a study of 208 palliative cancer patients in Chile, spiritual pain was associated with a reduced quality of life [45]. Quality of life in LATAM palliative care patients was influenced by the level of social and spiritual support they received [46]. While addressing spiritual pain can be important for palliative patients, there is little clinical guidance as to how this might be achieved [47]. It may be unreasonable to expect the clinical team to provide such spiritual comfort and it is not clear whether priests, rabbis, pastors, or social workers should be called. It is also not clear if clergy feel competent to address these situations where medicine and spirituality intersect.

In contrast to the United States and Western Europe, LATAM is deeply religious, and religious diversity is increasing. In 1960, about 90% of LATAM identified as Roman Catholic, but in 2020 LATAM was about 69% Roman Catholic, 19% Protestant, and 8% unaffiliated. Approximately 59% of Protestants and 30% of Catholics in palliative care participated in daily prayers, and weekly church services, and stated they had witnessed a miraculous healing [37, 48]. This does not include indigenous spiritual traditions, practices, and folk medicine, for which data are lacking.

In LATAM, curanderos are healers who practice herbalism and folk medicine, sometimes blended with spiritual practices. Even in the United States, Hispanics sometimes seek the services of these practitioners, mainly because they are affordable, reside in the community, are readily accessible, and offer empathy and culturally relevant spiritual support that contrasts with modern conventional medicine. In some cases, curanderos treat conditions that Western medicine does not even recognize [49]. In a survey of 3,728 adult Latinos residing in the United States, 6% reported they had consulted a curandero [50]. An important service offered by curanderos is the inclusion of cultural and spiritual values along with psychological reassurance [51]. In LATAM, the concurrent use of modern and folk medicine is common, although it is not unusual for a curandero’s client to fail to disclose such consultations to a medical doctor [52]. However, for patients with good prognoses, treatments from a curandero and a medical doctor are not necessarily in conflict. The patient may derive empathy and encouragement from the former and medical benefit from the latter. However, curanderismo appears to be incompatible with palliation. Curanderos strive for miracles and promote healing energies; both clients and curandero may feel that they have failed if their work does not produce a cure or at least improvement [51]. In some ways, curanderismo may help fuel therapeutic obstinacy, the desire to find a cure at any cost.

Social determinants can limit palliative care

Palliative care is not always considered an essential part of healthcare services in LATAM, particularly in areas that struggle to provide more basic, life-saving medical services. Yet palliation care is urgently needed because about two-thirds of all of the people who die of cancer die in a low-income or mid-income nation [53]. Universal healthcare plans do not necessarily include palliative services. In countries with mixed or open-market plans, palliative services may be available but only to those who can afford them.

Race and racial determinants of health are often eclipsed in public health and social science discussions of LATAM by social class. However, a new study suggests a complex and nuanced racial system where hair, skin color, and other physical characteristics sometimes act as surrogates for class [54]. The four main racial identities in LATAM are Black, white, indigenous (Indian), and mixed. These identities are based to a large degree on descent as well as physical characteristics [55]. Economic status appears to be a greater determinant of health than race in LATAM, but it may be that economic status is secondary to race or at least impacted by race [56]. Poverty is a greater social determinant of health in LATAM, but race may contribute to poverty [57]. Language barriers may be a further social determinant of health because there are numerous indigenous languages and some isolated groups may have only limited fluency in the national language [58].

An important determinant of health is whether residency is rural or urban. People living in rural communities may find access to home health workers limited or nonexistent; however, those living in poverty may find it impossible to pay for even basic medications or supplies even in cities. Racism itself may cause unequal access to care [59].

Equity in palliative care requires education into the social determinants of health, systemic healthcare biases, and a better understanding of the highly nuanced issue of class and racism in LATAM. An important recommendation for palliative care for all parts of the world is that the system represent the racial and ethnic populations it serves [59].

Vulnerable populations may also face discrimination in palliative care services in LATAM and other parts of the world. These groups include men who have sex with men (MSM), people with HIV/AIDS, and sexual minorities [60]. There is a paucity of studies on these particular populations with respect to palliation.

Low health literacy

In LATAM’s fragmented healthcare system serving diverse populations, health literacy is particularly needed so that individuals can determine the types of services they might need, evaluate offerings, navigate the system, and then make appropriate decisions based on the information they receive [61]. Neighboring nations in LATAM may have vastly different healthcare systems, so this is a national rather than regional concern.

Low health literacy results in individuals who do not get the care they need, even when that care is available, accessible, and affordable [58]. High health literacy gives empowered patients greater agency to direct the course of their own treatment and make informed decisions. In many ways health literacy is linked to social determinants of health; those with greater health literacy may be able to catapult over systemic inequities [58]. There are few studies on health literacy in LATAM.

In the case of palliative care, the individuals seeking information or making decisions may be family members rather than the patient. It takes a certain amount of health literacy to even know to seek palliation information. While a limited amount of such information may be found online, most would likely seek or obtain the information from healthcare professionals [62]. Healthcare professionals may not have time or awareness to volunteer the information unless directly asked.

Interventions to raise health literacy are generally needed and awareness campaigns to describe the role of palliation may help individuals know what services are available when confronting life-threatening illnesses. LATAM is far too large and diverse to capture in single statistics but estimates adequate health literacy from 5% to 73% [58]. 

Health literacy about palliation in LATAM is probably low because palliative care programs are relatively new and limited to certain urban settings. Thus, public awareness campaigns are going to have to play an important role in palliation. A challenge for palliative care is that there are different culturally driven understandings of palliation. That is to say, an urban dweller in Brazil may have different ideas about care for a person with a terminal illness than a rural resident in Brazil, and both may have distinctly different ideas than a person living in a small town in Guatemala. Moreover, palliation must encompass the difficult-to-discuss issues of decision-making at the end of life, and those decisions are likewise shaped by cultural attitudes about death, dying, and inheritance. Many patients seek to avoid these conversations, but palliative care sometimes forces them to occur. The role of caregivers, such as family members, and the medical establishment also come into play, and these are shaped by culture as well. In general, in LATAM family bonds are strong and family members will typically assume roles in caregiving and support, while the healthcare system likely will take a more distant but paternalistic role in medical decision-making [63].

Limited clinician training

Worldwide, more and better training for healthcare professionals in palliation is needed. Many clinicians first encounter palliative care in the clinic rather than in the classroom. Initial training in palliative care likely occurs in a collegial mentoring situation rather than an academic one. Few healthcare professionals in LATAM have any formalized training in palliative care [64]. Since palliation is counter-intuitive to many standard treatment philosophies, it can be startling or upsetting for clinicians to “surrender” their patients rather than implement more aggressive treatments [64]. Moreover, some patients and their families feel abandoned by their physicians when the subject of end-of-life is discussed.

More training programs are needed in LATAM both for clinicians and patients. A systematic review of the literature identified 36 palliative care training programs offered in eight LATAM nations. Most were postgraduate-level courses that focused on pain management [64]. In Chile, an advanced 12-day diploma course in palliative care was offered in a hybrid setting and assessments pre- and post-course demonstrated that completion of the course increased the healthcare workers’ knowledge of palliation and changed their behaviors as they offered these services [65]. Similar programs may be helpful across other nations.

A study evaluated palliative care education in 19 LATAM nations and reported that 42% of these countries offered palliative medicine as a distinct medical specialty, but 20% of these programs have not yet graduated their first class. These palliative care programs varied in length from two to four years or from 520 hours to three years when palliation was a subspecialty. Lack of adequate training is a formidable barrier to achieving adequate palliative services [66]. One barrier to more palliative care programs is the fact that palliation is not always reimbursed.

Hospitals and clinics encounter patients with grave diagnoses every day, yet many in LATAM have no specific protocols as to how to manage these cases. In a survey with follow-up semi-structured interviews, 82% of physicians and 69% of nurses at a single neurological center in Peru desired more and better training for palliative care, because the majority of respondents saw a need for palliation in at least 30% of patients [27].

Palliative care training programs can be assessed using the Efficacy in Palliative Care (SEPC) and Thanatophobia Scales [67]. A version of these scales was recently adapted and validated for the Portuguese language and use in Brazil [67]. In this survey, respondents who scored high on fear of death exhibited a negative correlation to self-efficacy in palliative care, that is, the more uncomfortable physicians felt dealing with death, the less confidence they exhibit in palliation [67].

Palliative care education is not part of the medical curricula in LATAM universities, although a survey of Brazilian physicians found 99% of respondents consider it an important topic for their clinical training. In this survey, the areas found most lacking in physician education were symptom management, particularly for pain, dyspnea, and mental health issues [68]. Thus, in LATAM there is a demand and need for greater palliative care education and training, but not necessarily a demand for making palliative care a medical specialty unto itself.

## Conclusions

Palliative care services are essential to modern healthcare and even recognized as a human right, particularly in view of prolonged longevity, the survivability of cancer, and changing approaches to holistic individualized care. However, how each nation implements palliation may be different and certainly each nation, as evidenced in LATAM, faces unique challenges as well as opportunities. The main challenges confronting LATAM palliation are universal and offer valuable insights and lessons about how palliative care can be better integrated into healthcare systems.
